# Visual and Colorimetric Sensing of Metsulfuron-Methyl by Exploiting Hydrogen Bond-Induced Anti-Aggregation of Gold Nanoparticles in the Presence of Melamine

**DOI:** 10.3390/s18051595

**Published:** 2018-05-17

**Authors:** Guangyang Liu, Ruonan Zhang, Xiaodong Huang, Lingyun Li, Naixin Liu, Jing Wang, Donghui Xu

**Affiliations:** 1 Quality and Safety Risk Assessment Laboratory for Sugar Crops Products, the Ministry of Agriculture, Heilongjiang University, Harbin 150080, China; liuguangyang@caas.cn; 2 Key Laboratory of Vegetables Quality and Safety Control, Institute of Vegetables and Flowers, Chinese Academy of Agricultural Sciences, Ministry of Agriculture of China, Beijing 100081, China; zrn704269188@163.com (R.Z.); huangxiaodong@caas.cn (X.H.); lilingyun@caas.cn (L.L.); 3 Key Laboratory of Agrifood Safety and Quality, Institute of Quality Standard and Testing Technology for Agro Products, Chinese Academy of Agricultural Sciences, Ministry of Agriculture of China, Beijing 100081, China; W_jing2001@126.com

**Keywords:** colorimetric sensor, gold nanoparticle, anti-aggregation, metsulfuron-methyl, melamine, agricultural irrigation water

## Abstract

Various highly sensitive and selective analytical methods have been used to monitor metsulfuron-methyl residue in the environment. However, these methods involve costly instruments and complex, time-consuming operations performed in laboratories. Here, a rapid, convenient, and sensitive colorimetric sensor based on anti-aggregation of gold nanoparticles (AuNPs) is demonstrated for the rapid detection of metsulfuron-methyl in agricultural irrigation water. The AuNPs could be induced to aggregate in the presence of melamine and exhibited a distinct color change from wine-red to blue. The aggregation was suppressed by a strong hydrogen-bonding interaction between metsulfuron-methyl and melamine. The differences of the absorbance at 523 nm (ΔA523) and the color change was linearly related to metsulfuron-methyl concentration over the range 0.1–100 mg/L, as observed visually and by UV-vis (Ultraviolet-visible) spectrometry. The detection limit of the sensor was as low as 0.05 mg/L (signal/noise = 3), and was used to determine metsulfuron-methyl in spiked water and in agricultural irrigation water samples. Recoveries were in the range of 71.2–100.4%, suggesting that the colorimetric sensor was suitable for the determination of metsulfuron-methyl in agricultural water samples.

## 1. Introduction

Metsulfuron-methyl is a systemic sulfonylurea pesticide that was introduced in the 1980s and has been widely used to control broad-leaved weeds and grasses in rice, maize, wheat, and barley [1]. Because of its highly efficient herbicidal activity and low mammalian toxicity, metsulfuron-methyl has been applied worldwide [2]. However, its high water solubility, high mobility, and slow degradation may result in contamination of soil, environmental water, and food. In addition, metsulfuron-methyl could bring harm to aquatic plants and animals and the overall ecosystem [3]. It is a great challenging task to monitor metsulfuron-methyl in environmental water and soil [4,5]. Hence, a highly sensitive, selective, and simple detection method is needed to trace low levels of metsulfuron-methyl in the environment, especially in aquatic systems. 

Various analytical methods have been used to detect metsulfuron-methyl residue in the environment, such as high performance liquid chromatography (HPLC) [1,6], gas chromatography (GC) [7], mass spectrometry (MS) [8], HPLC-MS [9], GC-MS [10], immunoassays [11], fluorimetry, and capillary electrophoresis [12]. These methods are highly sensitive and selective, but involve costly instruments and complex, time-consuming operations performed in laboratories. Therefore, a rapid, inexpensive, and highly sensitive analytical method is needed to determine metsulfuron-methyl in the field in real time.

Gold nanoparticle (AuNP) is a kind of noble metal nanoparticles which have received great attention because of their unique chemical, electronic, catalytic, and optical properties [13,14,15]. AuNPs can be induced to aggregate by various target analytes, with a corresponding color change from wine-red to blue derived from strong surface plasmon resonances [16,17,18]. The color change can be monitored visually or with a UV-vis (Ultraviolet-visible) spectrophotometer to quantitatively determine the amounts of chemical targets [19,20,21]. Many colorimetric sensors based on AuNP aggregation have been employed to detect pesticides residues, such as glyphosate [22], imidacloprid [23], atrazine [24], methyl parathion [25], and triadimenol [26]. However, heavy metal ions from soils, pigments found in fruits and vegetables, and organic molecules from complex matrices will disrupt the sensitivity and selectivity of AuNP aggregation colorimetry and produce false positives or incorrect detection results [27]. When compared with the above-mentioned AuNP aggregation colorimetry, the colorimetric sensor based on anti-aggregation of AuNPs has high sensitivity and selectivity, but can be subject to many interfering compounds [28,29,30,31,32,33,34]. Thus, few reports have used AuNPs anti-aggregation to monitor metsulfuron-methyl residue in aqueous samples.

Hence, a rapid, convenient, and sensitive colorimetric sensor based on AuNPs anti-aggregation was developed for the detection of metsulfuron-methyl in agricultural irrigation water. The AuNPs could be aggregated by melamine, with an apparent color change from wine-red to blue. However, strong hydrogen bonding between metsulfuron-methyl and melamine will prevent the aggregation of AuNPs. There was a linear relationship between 523-nm absorbance and metsulfuron-methyl concentration. Thus, the concentration could be quantitatively determined visually and by UV-vis spectrophotometry.

## 2. Materials and Methods

### 2.1. Chemicals and Apparatus

Metsulfuron-methyl, melamine, atrazine, and hexazinone were obtained from Sigma-Aldrich (St Louis, MO, USA). NaCl, MgCl_2_, glucose, L-cysteine, and vitamin C were purchased from Aladdin Industrial Corporation (Shanghai, China). Chloroauric acid (HAuCl_4_) was purchased from Sinopharm Chemical Reagent Co. Ltd. (Shanghai, China). All other reagents were analytical reagent grade.

A NanoDrop one^C^ spectrophotometer (Thermo Scientific, Waltham, MA, USA) was used to record UV-vis absorption spectra. A JEM-200CX transmission electron microscope (TEM, JEOL, Tokyo, Japan) was used for imaging particle aggregation. Vibrational spectra were obtained with a Fourier transform infrared spectrometer (FT-IR-8400, Shimadzu, Kyoto, Japan).

### 2.2. Synthesis of AuNPs

AuNPs were synthesized following the procedure reported previously with slight modifications [31,32]. All glassware was cleaned with aqua regia and rinsed with ultrapure water. An aqueous HAuCl_4_ solution (1 mM, 150 mL) was added to a 250-mL round-bottom flask and boiled for 30 min with vigorous stirring. Trisodium citrate solution (38.8 mM, 15 mL) was then added and the mixture was heated for another 15 min, exhibiting a color change from light yellow to wine-red. The wine-red solution was cooled and filtered through a 0.22 µm Millipore syringe filter, and the suspension was transferred to a stoppered conical flask and stored at 4 °C. 

### 2.3. Colorimetric Assay

Unless otherwise noted, the citrate-AuNP solution was used without further functionalization. An acetate buffer solution (10 mM, pH 3.5, 0.2 mL) was added to 0.2 mL of the AuNPs solution and incubated for 5 min (after incubation, the mixture solution provided low pH condition and melamine can be protonated in this situation and conjugated to the negatively-charged ions on the surface of AuNPs to induce its aggregation) [35]. Then, different concentrations of metsulfuron-methyl (0.1 mg/L, 0.2 mg/L, 0.5 mg/L, 1.0 mg/L, 2.0 mg/L, 5.0 mg/L, 10.0 mg/L, 20.0 mg/L, 50.0 mg/L, and 100.0 mg/L; 0.2 mL) were added and the mixtures were equilibrated for 5 min. Finally, melamine solution (2.0 mg/L, 0.1 mL) was added and the mixture was incubated for another 20 min. The color change was observed visually and the absorbance spectrum was monitored with the UV-vis spectrophotometer. The absorbance at 523 nm was linear to the logarithm of the metsulfuron-methyl concentration and then was used to estimate the detection response. The sensor selectivity was tested with atrazine, hexazinone, glucose, L-cysteine, vitamin C, Na^+^, and Mg^2+^ as potential interfering substances at the same concentrations as those of metsulfuron-methyl (20 mg/L, 10 mg/L, 5 mg/L, and 2 mg/L). 

### 2.4. Sample Pretreatment

Tap and agricultural irrigation water samples were mixed with ethylenediaminetetraacetic acid (EDTA, 1 mM) to remove heavy metal ions, and the solution was then filtered through a 0.2 µm Millipore syringe filter. The water samples were spiked with different metsulfuron-methyl concentrations, and the colorimetric sensor was used to quantitatively determine the concentration. 

## 3. Results

### 3.1. Characterization

**Figure 1:**
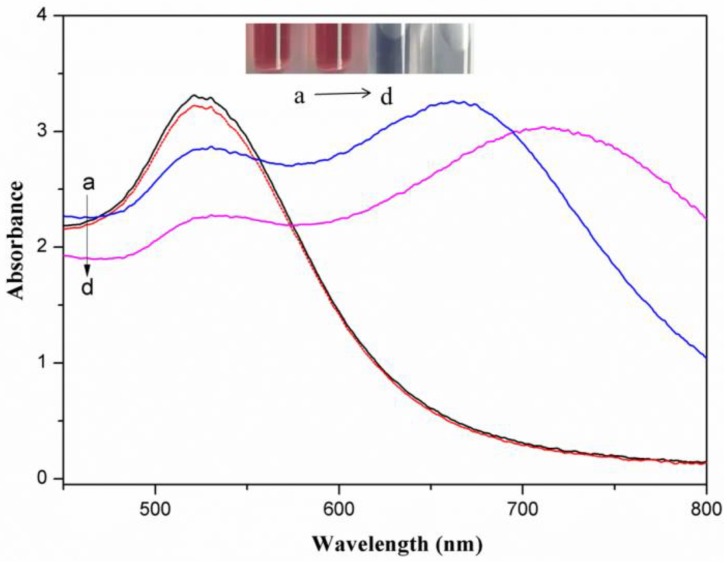
(a → d) Surface plasma resonance absorbance spectra for gold nanoparticles (AuNPs), AuNPs with 2.0 mg/L metsulfuron-methyl, AuNPs with 2.0 mg/L metsulfuron-methyl and 2.0 mg/L melamine, and AuNPs with 2.0 mg/L melamine, respectively (acetate buffer solution: 10 mM, pH 3.5, 0.2 mL). Insert: picture of AuNPs solutions.

Figure 1 presents surface plasma resonance absorbance spectra for AuNPs, AuNPs with 2.0 mg/L metsulfuron-methyl, AuNPs with 2.0 mg/L melamine, and AuNPs with 2.0 mg/L metsulfuron-methyl and 2.0 mg/L melamine. The unmodified AuNPs possess a distinct wine-red color and a strong absorption peak at 523 nm. The color of AuNPs remained wine-red after adding metsulfuron-methyl. When the AuNPs were aggregated by melamine, the color changed to blue, the 523-nm adsorption decreased, and a new absorption band appeared at 710 nm. However, when the mixture of AuNPs and metsulfuron-methyl was incubated with melamine, much less aggregation occurred, and the color changed slightly or remained purple if there was a high concentration of metsulfuron-methyl. 

**Figure 2:**
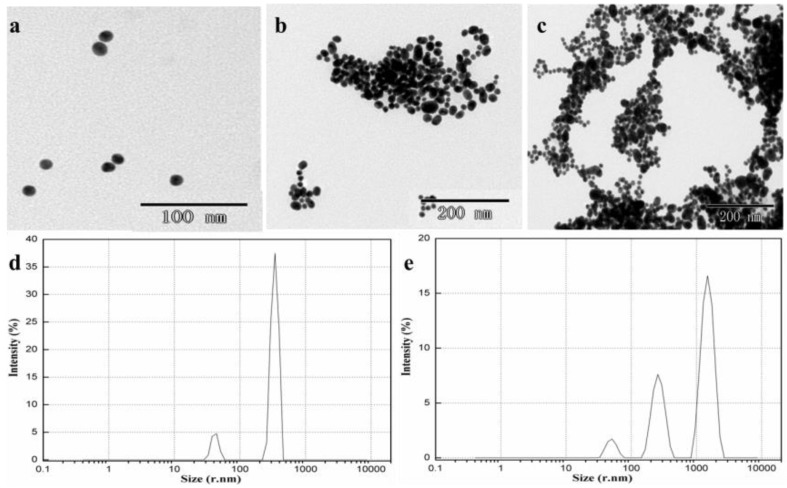
Transmission electron microscope (TEM) images of (**a**) AuNPs with 2.0 mg/L metsulfuron-methyl; (**b**) AuNPs with 2.0 mg/L metsulfuron-methyl and 2.0 mg/L melamine; (**c**) AuNPs with 2.0 mg/L melamine, the particle size distributions images of (**d**) AuNPs with 2.0 mg/L metsulfuron-methyl and 2.0 mg/L melamine; and (**e**) AuNPs with 2.0 mg/L melamine (acetate buffer solution: 10 mM, pH 3.5, 0.2 mL).

Figure 2 shows TEM images of microstructures of AuNPs with 2.0 mg/L metsulfuron-methyl, AuNPs with 2.0 mg/L metsulfuron-methyl and 2.0 mg/L melamine, and AuNPs with 2.0 mg/L melamine. AuNPs with 2.0 mg/L metsulfuron-methyl exhibited monodispersity and a uniform size, around 20 nm (Figure 2a), which suggested that metsulfuron-methyl could not induce AuNPs to aggregate. Only in the presence of melamine, the AuNPs were induced to aggregate, as shown in Figure 2c. After adding metsulfuron-methyl to the AuNP-melamine mixture, fewer and smaller aggregations were present (Figure 2b) than for AuNPs with 2.0 mg/L melamine, indicating that the complex formation between metsulfuron-methyl and melamine inhibits the aggregation. 

**Figure 3:**
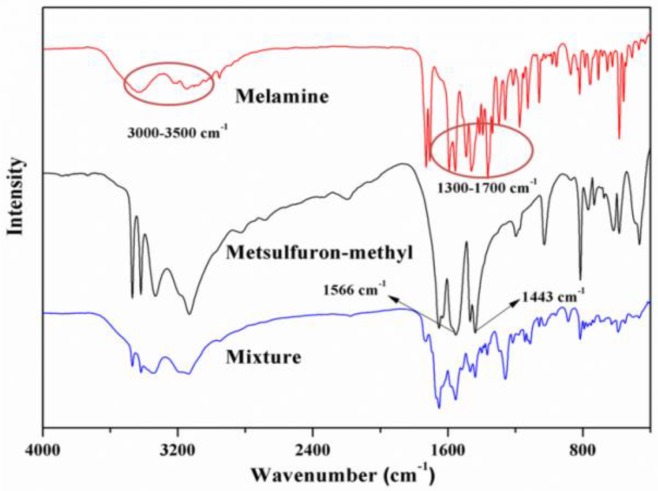
Fourier transform infrared spectrometer (FT-IR) spectra of the following: melamine, metsulfuron-methyl, and the complex of melamine and metsulfuron-methyl.

The hydrogen bonding interaction between metsulfuron-methyl and melamine was examined with FT-IR spectroscopy. As shown in Figure 3, the spectra of metsulfuron-methyl had characteristic peaks for –NH– at 1566 cm^−1^ and for –R–SO_2_– stretching at 1443 cm^−1^. In the spectrum of melamine, adsorption peaks at 3000–3500 cm^−1^ for the –NH_2_ group and 1300–1700 cm^−1^ for the benzene ring substitution group (–N=CH–) were observed. In the metsulfuron-methyl-melamine complex, the –NH_2_ peak was shifted and the intensities of the stretching vibrations of –R–SO_2_– in metsulfuron-methyl and –N=CH– in melamine decreased. These observations indicated that the hydrogen bonding interaction between metsulfuron-methyl and melamine was formed.

### 3.2. Colorimetric Sensing Mechanism 

**Figure 4:**
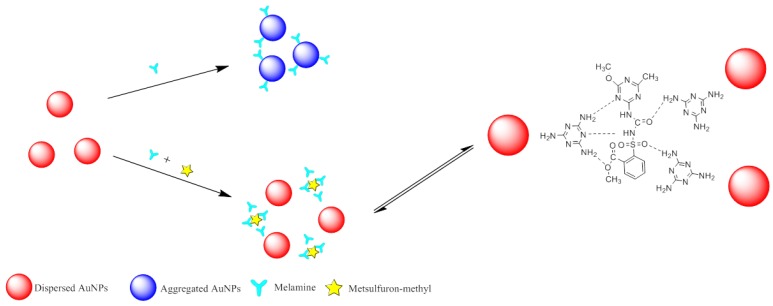
Schematic illustration of colorimetric sensing of metsulfuron-methyl based on anti- aggregation of gold nanoparticles in the presence of melamine.

Figure 4 illustrates the mechanism of the colorimetric sensor for metsulfuron-methyl detection based on anti-aggregation of AuNPs induced by melamine. The wine-red AuNP solution exhibited a strong SPR absorption peak at 523 nm. The AuNPs could be aggregated by melamine, with an accompanying color change to blue. However, the combination between metsulfuron-methyl and melamine will generate the strong hydrogen bonding interaction to prevent the aggregation of AuNPs. Thus, melamine and metsulfuron-methyl were used as the aggregation and anti-aggregation reagents, respectively, in this colorimetric sensing assay. Because there was a linear relationship between the 523-nm absorbance and the metsulfuron-methyl concentration, the concentration of metsulfuron-methyl could be qualitatively determined.

### 3.3. Optimization of Method

#### 3.3.1. Effect of Melamine Concentration

**Figure 5:**
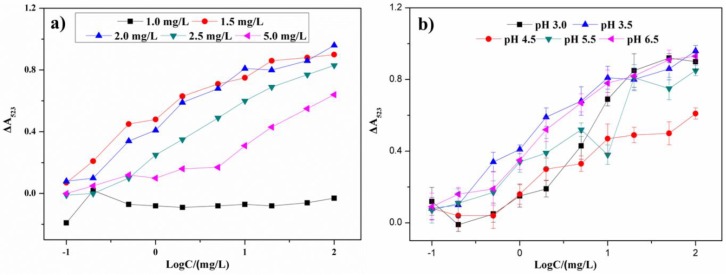
The plots of ΔA523 versus metsulfuron-methyl concentration under (**a**) different concentration of melamine and (**b**) different pH conditions (pH 3.0, pH 3.5, pH 4.5, pH 5.5, and pH 6.5).

The optimal concentration of melamine was an important factor for the AuNP aggregation and to affect the sensitivity and linear range of the colorimetric sensor. In Figure 5a, with the concentration of melamine increasing, an increase of the absorbance ratio in the presence of different concentrations of metsulfuron-methyl is observed. The absorbance difference ΔA523 was equal to (A_blank_ − A_sample_), where A_blank_ was the absorbance without metsulfuron-methyl and A_sample_ was the absorbance with metsulfuron-methyl. When the melamine concentration was above 2.0 mg/L, the level of AuNP aggregation was too high and the analytical signals decreased and narrowed the linear range of the sensor. When the concentration was below 2.0 mg/L, the analytical signals became less sensitive. Therefore, 2.0 mg/L was the optimum melamine concentration used in experiments discussed below. 

#### 3.3.2. Effect of pH

Because the pH can affect the stability and the sensitivity of this sensor, an acetate buffer solution was varied over pH 3.0–6.5. As shown in Figure 5b, when the pH was above 3.5, the AuNPs were less sensitive to melamine and the limit of detection increased. When the pH was less than 3.5, the AuNPs were easily aggregated, which decreased the anti-aggregation performance of metsulfuron-methyl. Therefore, pH 4.5 was chosen as the optimum pH for experiments discussed below.

### 3.4. Colorimetric Sensing of Metsulfuron-Methyl

**Figure 6:**
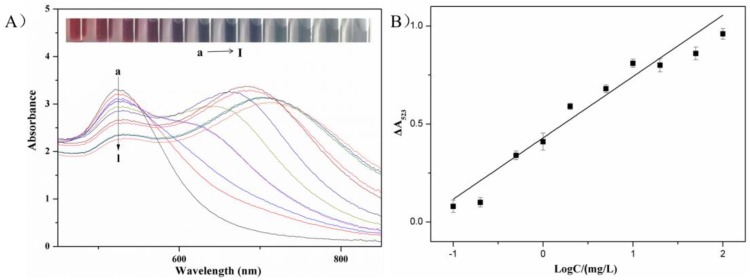
(**A**) (a → l) UV-Vis spectra of AuNP solutions, AuNP solutions with concentrations of metsulfuron-methyl of 100 mg/L, 50 mg/L, 20 mg/L, 10 mg/L, 5.0 mg/L, 2.0 mg/L, 1.0 mg/L, 0.5 mg/L, 0.2 mg/L, 0.1 mg/L, and 0 mg/L in the presence of 2.0 mg/L melamine. Inset: photographic images of AuNPs solutions with various concentrations of metsulfuron-methyl. (**B**) Standard calibration curve of the absorbance change of AuNPs at 523 nm (ΔA523) against metsulfuron-methyl concentration.

Metsulfuron-methyl concentrations over the range 0.1–100 mg/L were used to investigate the limit of detection and the linear range of the colorimetric sensor. Figure 6A revealed that the wine-red AuNPs solution was aggregated by melamine, producing a new adsorption peak at 640 nm and reducing the intensity of the 523-nm surface plasmon resonance adsorption. The aggregation was inhibited when metsulfuron-methyl was initially added to the AuNPs solution. The color changed from blue (purple) to wine-red, the 640-nm peak intensity decreased, and the 523-nm peak intensity increased.

The calibration linearity was evaluated by measuring ΔA523 vs. the log of the metsulfuron-methyl concentration. The curve fit in Figure 6B indicated a linear relationship over the range 0.1–100 mg/L. The calibration fit was ΔA523 = 0.305*x* + 0.4105 (*x* = log (conc.)) with a correlation coefficient *R*^2^ = 0.9568. The detection limit (signal/noise (*S*/*N*) = 3) was 0.05 mg/L. In Table 1, the analytical performance of the sensor was compared with previously reported metsulfuron-methyl detection methods. Relative to these methods, the colorimetric sensor was more rapid, convenient, and sensitive without requiring sophisticated instruments or time-consuming treatment procedures. 

### 3.5. Selectivity

**Figure 7:**
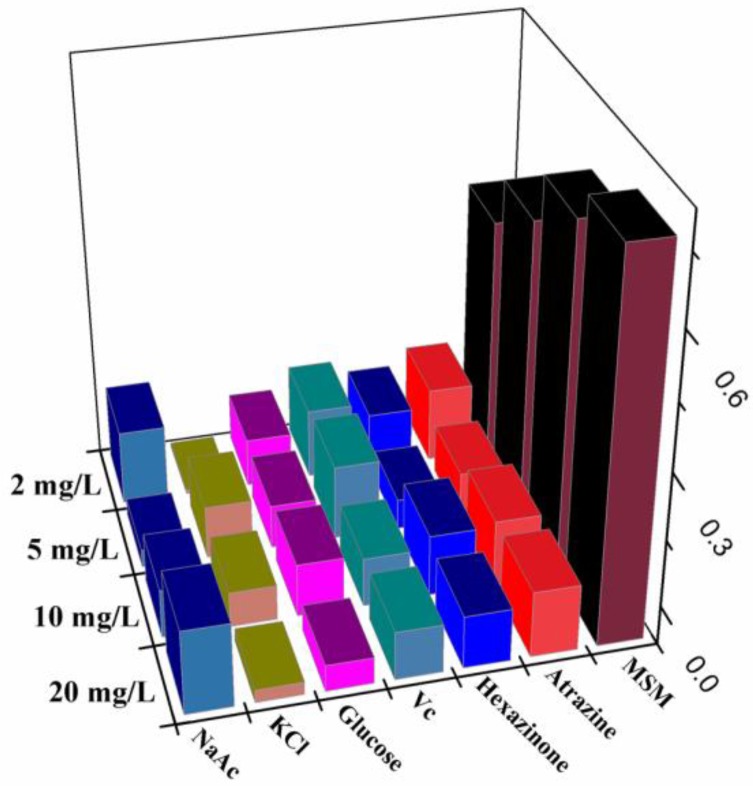
Absorption change ΔA523 of AuNPs in the presence of metsulfuron-methyl (MSM) and other interfering substances.

In the selectivity experiments, atrazine and hexazinone were chosen as the structure analogue for metsulfuron-methyl to study the anti-interference capacity of this sensor. Additionally, glucose, vitamin C, Na^+^, and Mg^2+^ were used as potential organic and inorganic interference compounds. Their concentrations (20 mg/L, 10 mg/L, 5 mg/L, and 2 mg/L) were the same as those used above for metsulfuron-methyl. As shown in Figure 7, only metsulfuron-methyl inhibited the aggregation of AuNPs by melamine and prevented the color change to blue. None of the above compounds exhibited anti-aggregation and no spectral changes were observed. Therefore, the colorimetric sensor had high selectivity and responsiveness to metsulfuron-methyl. 

### 3.6. Application to Agricultural Irrigation Water

To validate the analytical performance of the colorimetric sensor, metsulfuron-methyl was added to tap water and to agricultural irrigation water samples (containing 1 mM EDTA), and was then analyzed under the optimized procedure. The results in Table 2 reveal metsulfuron-methyl recoveries of 0.5 mg/L, 1.0 mg/L, and 5.0 mg/L from spiked tap water samples that ranged from 74.3–93.1%, respectively, with relative standard deviations (RSDs) that ranged from 4.73–6.37%. Metsulfuron-methyl recoveries of 0.5 mg/L, 1.0 mg/L, and 5.0 mg/L from spiked well water samples ranged from 71.2–100.4%, and RSDs ranged from 3.81–6.52%. These results suggest that the colorimetric sensor was suitable for the determination of metsulfuron-methyl in agricultural water samples.

## 4. Conclusions

A rapid, highly sensitive, and selective colorimetric sensor to monitor metsulfuron-methyl residue in agricultural irrigation water samples was demonstrated. The sensing mechanism was based on preventing melamine-induced aggregation of AuNPs by strong hydrogen bonding between metsulfuron-methyl and the melamine. The mechanism was verified via FT-IR and UV-Vis spectra, and TEM imaging. Under optimum experiments conditions, the ΔA523 were found to be linearly related to different metsulfuron-methyl concentration (0.1 to 100 mg/L). The detection limit was as low as 0.05 mg/L (*S*/*N* = 3). The colorimetric sensor was performed easily and economically with no need for complex experimental procedures or instruments. All experimental steps took no more than 30 min to finish. Its rapid response, high sensitivity, and good test sample recoveries indicate that it has applicability for metsulfuron-methyl residue detection in aqueous water samples.

## Figures and Tables

**Table 1:** Comparison of the linear ranges and detection limits of different methods.

Methods	Linear Range (mg/L)	LOD (mg/L)	*R* ^2^	Matrices	Reference
HPLC-DAD	0.2–5	0.03	0.999	Soya milk	[1]
HPLC-UV	0.05–5	0.011	0.999	Soil	[6]
Immunoassay	-	0.005	0.990	Environmental water	[11]
DTC-PTBCA-AuNPs based UV-vis detection	0.381–19.1	0.0724	0.996	Environmental water	[19]
Anti-aggregation-AuNPs based UV-vis detection	0.1–100	0.05	0.957	Irrigation water	This work

**Table 2:** Recoveries of metsulfuron-methyl in water samples (*n* = 3).

Samples	Spiked (mg/L)	Found (mg/L)	Recovery (%)	RSD (%)	LOD (mg/L)
Tap water	0	-	-	-	0.25
	0.5	0.372	74.3	6.37	
	1.0	0.852	85.2	5.46	
	5.0	4.66	93.1	4.73	
Well water	0	-	-	-	0.5
	0.5	0.356	71.2	5.72	
	1.0	0.886	88.6	3.81	
	5.0	5.02	100.4	6.52	
